# Laser writing of metal-oxide doped graphene films for tunable sensor applications†

**DOI:** 10.1039/d4na00463a

**Published:** 2024-12-10

**Authors:** Shasvat Rathod, Monika Snowdon, Matthew Peres Tino, Peng Peng

**Affiliations:** a Centre for Advanced Materials Joining, Department of Mechanical and Mechatronics Engineering, University of Waterloo 200 University Avenue West Waterloo Ontario N2L 3G1 Canada peng.peng@uwaterloo.ca

## Abstract

Flexible and wearable devices play a pivotal role in the realm of smart portable electronics due to their diverse applications in healthcare monitoring, soft robotics, human–machine interfaces, and artificial intelligence. Nonetheless, the extensive integration of intelligent wearable sensors into mass production faces challenges within a resource-limited environment, necessitating low-cost manufacturing, high reliability, stability, and multi-functionality. In this study, a cost-effective fiber laser direct writing method (fLDW) was illustrated to create highly responsive and robust flexible sensors. These sensors integrate laser-induced graphene (LiG) with mixed metal oxides on a flexible polyimide film. fLDW simplifies the synthesis of graphene, functionalization of carbon structures into graphene oxides and reduced graphene oxides, and deposition of metal-oxide nanoparticles within a single experimental laser writing setup. The preparation and surface modification of dense oxygenated graphene networks and semiconducting metal oxide nanoparticles (CuO_*x*_, ZnO_*x*_, FeO_*x*_) enables rapid fabrication of LiG/MO_*x*_ composite sensors with the ability to detect and differentiate various stimuli, including visible light, UV light, temperature, humidity, and magnetic fluxes. Further, this *in situ* customizability of fLDW-produced sensors allows for tunable sensitivity, response time, recovery time, and selectivity. The normalized current gain of resistive LiG/MO_*x*_ sensors can be controlled between −2.7 to 3.5, with response times ranging from 0.02 to 15 s, and recovery times from 0.04 to 6 s. Furthermore, the programmable properties showed great endurance after 200 days in air and extended bend cycles. Collectively, these LiG/MO_*x*_ sensors stand as a testament to the effectiveness of fLDW in economically mass-producing flexible and wearable electronic devices to meet the explicit demands of the Internet of Things.

## Abstract 2

This study presents affordable fiber laser direct writing of durable and flexible, graphene-metal nanoparticle sensors with highly tunable responses.

## Graphical abstract

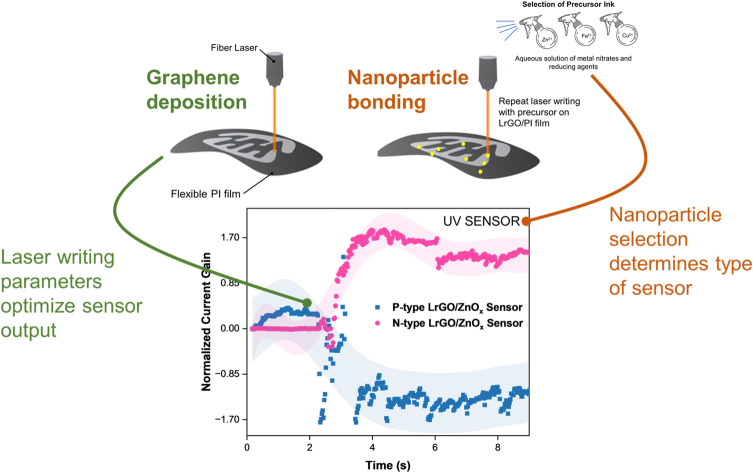

## 1 Introduction

Wearable electronics play a pivotal role in seamlessly integrating us and our environment through flexible sensor systems and wireless connectivity. Over the past decade, substantial efforts have been dedicated to creating versatile human-interactive devices that mimic skin-like functions, such as sensing touch, humidity, or temperature.^1–4^ While traditional planar integrated-circuit devices are sophisticated, their rigidity and fragility render them unsuitable for the soft and curvilinear forms of the human body. In contrast, flexible wearable electronics, which are bendable and adapt to the body without causing discomfort, enable a broad spectrum of sensing functionalities.^1,5,6^ In recent years, nanostructures composed of metal oxides (MO), including Co_3_O_4_, Cu_2_O, Fe_2_O_3_, In_2_O_4_, TiO_2_, SnO_2_, WO_3_, and ZnO, have gained widespread attention in sensing due to their exceptional chemical stability, mechanical flexibility, and substantial specific surface area.^7–10^ These metal oxide nanoparticles are particularly favored for applications in light sensing and gas sensing because they are biosafe, biodegradable, and biocompatible.^4,8,9^ Furthermore, high-performance room temperature sensors are also possible by hybridizing metal oxides with graphene to enhance charge transport.^11^

Likewise, graphene oxide (GO), stands out for its rich content of oxygen functionalities, rendering it electronegative and facilitating the fixation of metal cations on its surface through electrostatic interaction or chemical bonding.^7,12^ Additionally, the rigid surface of GO serves as an ideal substrate for nucleation, crystal growth, and the formation of ultrafine nanostructures with homogeneous dispersion and controlled morphologies.^12–14^ The presence of oxygen functional groups on the material surface enhances the electron and charge transfer rate, rendering GO water-soluble and biocompatible.^15^ Upon reduction, GO transforms into reduced graphene oxide (rGO), retaining some residual oxygen and structural defects. rGO exhibits remarkable thermal conductivity comparable to doped conductive polymers,^16^ approximately 36 times higher than Si and roughly 100 times higher than GaAs.^17^ Furthermore, rGO provides tunable electrical properties for specific applications through the functionalization of oxygen groups. Consequently, rGO holds tremendous potential as a cost-effective alternative to Si and metal-based sensors.^18^ However, despite its promising attributes, the practical application of rGO sensors is currently confined to research laboratories and has not yet transitioned to the market.^15,19,20^ One of the challenges dragging this transition is the rapid, cost-effective, and large-scale industrial production of these sensors.^21^ Currently, the conventional methods for producing rGO provide limited yield of graphene oxide. Many sensors incorporating rGO composites predominantly rely on electrochemical methods, which, though effective, present challenges in terms of cost and environmental longevity.^8,9^ A seamless process to combine rapid GO/rGO film fabrication with metal oxide nanoparticle deposition on flexible substrates can revolutionize the mass production of wearable flexible sensors.

In this investigation, a simple fiber laser direct writing (fLDW) process is employed to produce flexible graphene sensors with MO nanoparticles. fLDW is a one-step consolidation of multiple printing processes.^9^ This singular process can effectively (i) synthesize graphene, (ii) optimize the performance of carbon structures through functionalization, (iii) perform p– or n–doping and hybridization, and (iv) deposit metal-oxide nanoparticles. The variation of laser writing parameters allows for the modification of the LiG surface, its functional group concentrations, and MO nanoparticle concentrations, thereby elevating, or obstructing the electrical and optoelectronic properties of the LiG/MO_*x*_ sensors. Consequently, the fabricated LiG/MO_*x*_ sensors offer exceptional controllability in selectivity, response time, recovery time, reproducibility, and stability. The formation of LiG/MO_*x*_ sensors and their performance will also be evaluated by in-depth characterizations. As a proof of concept, flexible sensor responses to various elemental stimuli, such as temperature gradients, visible light, UV light, magnetic fluxes, and humidity will be demonstrated.

## 2 Materials and methods

Kapton® was purchased from McMaster-Carr. The polymer is produced through a polycondensation reaction between pyromellitic dianhydride (PMDA) and 4,4′-diaminodiphenyl ether (ODA). The resulting imide groups (–CO–N–CO–) in the polymer structure enable laser irradiation to convert the polyimide into graphene and graphene oxide compounds.

The precursor ink was prepared following the procedure by Rathod *et al.*^22,23^ This process involved dissolving 2 M polyethylene glycol (PEG, Sigma Aldrich, 8000 mol. wt., 99% purity), 2 M polyvinylpyrrolidone (PVP, Sigma Aldrich, ∼110 μm particle size, mol. wt. 120, 99% purity), and 2 M copper nitrate (Sigma Aldrich, trihydrate, mol. wt. 241, 99% purity) or 2 M zinc nitrate hexahydrate (Sigma Aldrich, mol. wt. 297.49, 99% purity) or 2 M iron nitrate (Sigma Aldrich, nonahydrate, mol. wt. 404, 99% purity) in deionized (DI) water. The MO_*x*_ solutions are made in volumetric ratios of 5 : 1 : 1 : 3 with deionized water, polyethylene glycol 0.2 g mL^−1^, polyvinyl pyrrolidone 0.1 g mL^−1^, and respective metal nitrates (copper nitrate, zinc nitrate, and iron nitrate). The solution was sonicated for 15 minutes until homogeneity. Deposition of the MO_*x*_ nanoparticle solution, onto the PI substrate, was achieved through a spray pump bottle. The DynalonTM Quick MistTM HDPE Sprayer bottles provide a fine mist of roughly 1/22 mL, sprayed from a distance of 15 cm from the PI substrate.

fLDW was performed using a YLR-30-MM-AC IPG continuous wavelength fiber laser within a MIYACHI MX2000 Glovebox. The laser, operating at 1070 nm, has a maximum output power of 30 W and a spot diameter of 500 μm.

The fabricated materials underwent comprehensive electrical and optoelectronic characterization using a Keithley 4200A semiconductor parameter analyzer (Tektronik, Beaverton, OR USA). The morphologies and compositions of the induced patterns were assessed through scanning electron microscopy (SEM, Zeiss FESEM 1530, Carl Zeiss, Munich, Germany) equipped with energy dispersive spectroscopy (EDS, VGS ESCALab, ThermoFischer Scientific). For sensor response tests under light and UV light conditions, a Dicuno© 5 mm light-emitting diode, “warm blue”, emitting at a wavelength of 625 nm, with electrical properties of 20 mA, 3–3.2 V, and 12 000–32 000 mcd was utilized. Additionally, a red LED emitting at a wavelength of 700 nm, with electrical properties of 20 mA, 2–2.2 V, and 2000–3000 mcd, and a UV LED at a wavelength of 395 nm, with electrical properties of 20 mA, 3–3.2 V, and 600–800 mcd were employed for the tests. For sensor response tests related to magnetic field fluxes, two standard ring magnets with a radius (*r*) of 0.05 m, width (*w*) of 0.03 m, remanence (Br) of 1.06 T, and a calculated magnetic flux density of 10^−4^ T at a distance of 5 cm from the sensor were used. Humidity tests were conducted in a humidity chamber set at 95% humidity. Bend tests were performed with a NEMA 12-step motor and a 3D printed holder with a bend radius of 4.02 mm and strain of 0.7% for the monolayer model. X-ray photoelectron spectroscopy (XPS, VGS ESCALab 250 Imaging ESCA, Thermo Fischer Scientific, Waltham, MA USA) was performed, and data were analyzed using CASAXPS. X-ray diffraction (XRD, PANalytical X'pert Pro MRD HR-XRD, Malvern Panalytical, Malvern UK) was conducted and analyzed using Match! Software (Crystal Impact, Bonn, Germany) using a standard 2Theta-omega scan from 10–90°.

## 3 Results and discussion

### 3.1 Composition of LiG/MO_*x*_ films

**Fig. 1:**
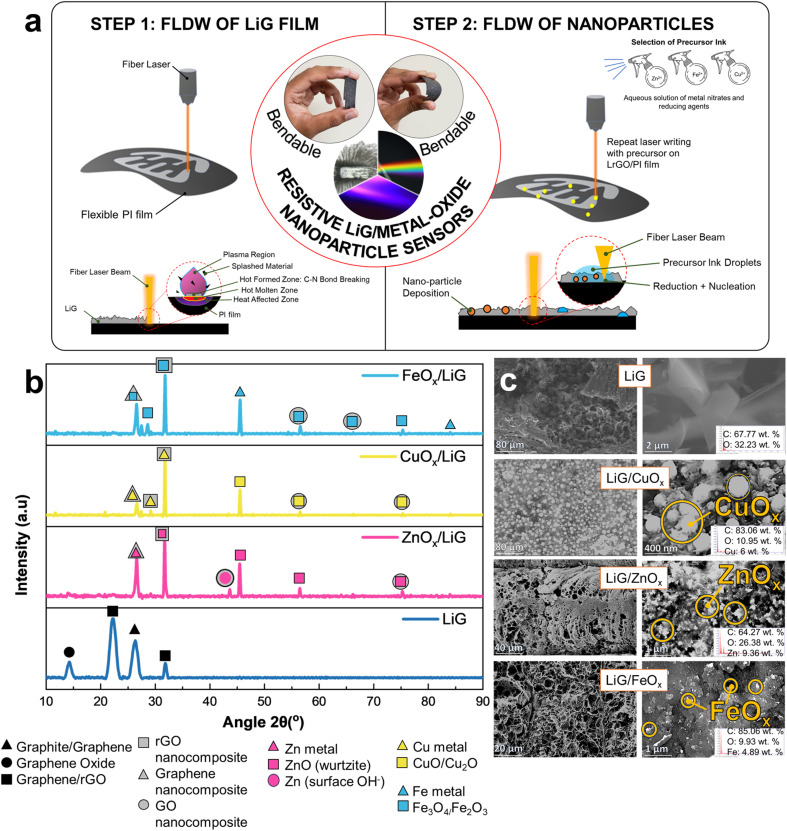
(a) Fabrication mechanism and schematic of LiG/MO_*x*_ sensor with fLDW. (b) XRD spectra analysis and (c) SEM images with EDS results of LiG/MO_*x*_ films.

Fig. 1a illustrates the fabrication procedure employed for the production of flexible LiG/MO_*x*_ nanoparticle sensors on polyimide (PI) substrates, adapted from Rathod *et al.*^22,23^ First, the PI film undergoes irradiation/ablation using a fiber laser to generate porous LiG films – the application of high laser energy density during laser ablation results in the cleavage of C–O, C=O, and C–N bonds.^15^ Subsequently, the newly discharged carbon atoms rearrange to form a graphene structure or gases. Furthermore, fLDW under ambient atmospheres can introduce oxygen functional groups on the induced graphene structures.^22^ As a result, the porosity, density, and conductivity nature of LiG are adjustable by varying the laser parameters. Second, the precursor ink is sprayed onto the newly formed LiG films, followed by another round of laser scanning. Cu(NO_3_)_2_, Fe(NO_3_)_2_, and Zn(NO_3_)_2_ are used in the precursor ink interchangeably to deposit CuO_*x*_, FeO_*x*_, and ZnO_*x*_ nanoparticles, respectively. Similar to the LiG film, and well-documented, the composition of the metal/metal oxide deposits can be modified by varying laser processing parameters.^10,15,24–26^

XRD analyses presented in Fig. 1b show the structural characteristics of LiG films, revealing their amorphous nature with discernible phases of small graphite, graphene oxide, and reduced graphene oxide. Specifically, a prominent peak at 2*θ* = 26.62° along the (002) plane is observed for graphite.^27^ The peak at 2*θ* = 14.5° indicates partial oxidation of graphite phases to GO.^27–29^ Additionally, broader peaks at 2*θ* = 24.10° signify the presence of rGO.^19,30^ Furthermore, a diffraction peak is observed at 2*θ* = 32°, which is caused by the reduction of graphene oxides.^31^ On the other hand, in the XRD spectra for LiG/MO_*x*_ films, the characteristic peak of graphene oxide at 2*θ* = 14° is notably absent, replaced by sharper graphite and rGO oxide peaks, particularly a sharper peak corresponding to graphite/rGO at 2*θ* = 32°. Generally, a greater intensity of the 32° peak indicates a fully graphitic system.^32^ This observation suggests that during the initial laser pass, fLDW predominantly deposits GO, which undergoes treatment to form rGO/Gr after secondary passes^31,33^ due to a precipitation reaction with metal ions from the precursor ink.^33^ Thus, fLDW can deposit multiple forms of graphite, graphene, graphite oxides and graphene oxides.

In the case of LiG/ZnO_*x*_ films, a characteristic rGO/ZnO nanocomposite peak at 2*θ* = 32° is observed, alongside a Gr/Zn phase peak at 2*θ* = 26°.^34^ Furthermore, three distinct diffraction peaks are present, separate from LiG peaks, at 43.5°, 45.5°, and ∼56°. First, an additional non-characteristic peak at ∼44° could be a product of GO/Zn-metal composites or symptomatic of the presence of surface hydroxyls in ZnO; a byproduct of fLDW in ambient air conditions.^35^ Second, peaks at 2*θ* = 45.5° and 2*θ* = 56.5° are characteristic of a hexagonal wurtzite structure of ZnO at planes (102) and (110), respectively. A minor peak is present highlighting the presence of GO/ZnO nanocomposites at 2*θ* = 75.5°. The calculated composition breakdown through Rietveld refinement is 93 at. % ZnO and 7 at. % metallic Zn (JCPDS: 36-1451).^36,37^

Similarly, CuO_*x*_ nanoparticle deposition on LiG showed mixed Gr/CuO/Cu_2_O phases at 2*θ* = (26°, 27°, 29°, 32°, 44°, and 56°). The peaks at 2*θ* = 26° and 2*θ* = 27° correspond to the presence of carbon/copper-oxide composites with graphene and rGO phases, respectively.^38,39^ Notably, the graphene peak at 2*θ* = 26° is significantly less defined for CuO_*x*_ than ZnO_*x*_ depositions, suggesting a lower weight ratio of graphene to copper than graphene to zinc.^34^ This discrepancy may arise from the fLDW process, wherein the metal and metal-oxide nanoparticles are induced by reducing ions present in the precursor solution. Because copper has a higher reduction potential compared to zinc, it tends to be induced more readily. Consequently, this leads to a lower ratio of graphene to copper in the resulting composite material. Peak 2*θ* = 32°, as aforementioned, is indicative of rGO/metal–Cu phases in the LiG/CuO_*x*_ film.^40^ All discernible peaks associated with copper indicate the polycrystalline nature of the resulting product.^41^ Specifically, the distinct cuprous oxide peak at 2*θ* = 29° and the metallic fcc-Cu peak at 2*θ* = 44° align with the (110) and (111) phases, respectively.^42,43^ Furthermore, minor but discernible peaks at 2*θ* = 56.5° and 2*θ* = 74.5° are observed in GO/CuO nanocomposite structures.^44^ Rietveld refinement through Match! Calculates a composition of 53.8 at. % CuO, 24 at. % Cu_2_O, and 22.2 at. % Cu (JCPDS 04-0836).^45,46^

Likewise, LiG/FeO_*x*_ XRD analysis indicates the presence of mixed graphene and Fe metal, Fe_3_O_4_, Fe_2_O_3_/FeO peaks at 2*θ* = (26°, 27°, 29°, 32°, 46°, 56°, and 75°). In the case of nanoparticles, it is difficult to distinguish between magnetite Fe_3_O_4_ and maghemite γ-Fe_2_O_3_ phases, solely from XRD. Peaks corresponding to Gr/FeO_*x*_ and rGO/Fe_3_O_4_ nanocomposites are evident at 2*θ* = 26° and 2*θ* = 32°, respectively.^47–50^ At 2*θ* = ∼27–29°, a characteristic rhombohedral hematite α-Fe_2_O_3_ phase was observed, previously found to appear in GO/FeO_*x*_ nanocomposites annealed at extremely high temperatures.^49^ A characteristic iron peak at 2*θ* = 46° is clearly present.^51^ Whereas weaker peaks are observed at 56°, 66°, 74°, and 84°; denoting GO/FeO_*x*_ nanocomposite, GO/Fe_3_O_4_ nanocomposite, magnetite Fe_3_O_4_, and Fe phases, respectively.^34,48–52^ The smaller Fe_2_O_3_ peaks imply fLDW deposition through the reduction of Fe ions to Fe_2_O_3_ magnetic nanoparticles (MNPs) to further reductions into Fe_3_O_4_.^53^ Rietveld refinement confirms nanoparticle composition of 91.5 at. % Fe_3_O_4_ and 8 at. % Fe (JCPDS No. 75-0033, 39-1346, 90-2377).^54^

Overall, the XRD analysis provides compelling evidence of graphene and nanoparticle deposition and graphene-metal/MO bonding. However, the presence of multiple phases of graphene and various phases of selected nanoparticles complicates the precise determination of their exact states. SEM and EDS results summarized in Fig. 1c highlight the presence of metal and metal oxide nanoparticle depositions after step two of fLDW ranging in size and localized concentrations. Visually, there is a pronounced affinity of metal nucleation at defect sites.^55–57^ At low magnifications, concentrated NP deposits are observed at the porous edges of the LiG film.

### 3.2 Effects of p-type and n-type LiG films

**Fig. 2:**
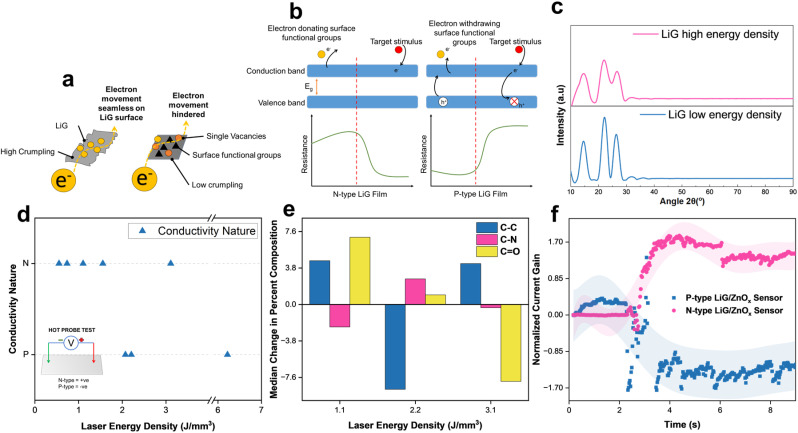
(a) Schematic of electron transport modification on LiG film. (b) Resistive metal-oxide sensing mechanism for p-type and n-type LiG. (c) XRD spectra of GO and rGO films produced through fLDW (d) Laser energy density *vs.* Conductivity nature of induced LiG films. (e) Laser energy density *vs.* change in functional groups, data educed from XPS C 1s scans. (f) Time *vs.* normalized current gain of P-type and N-type LiG/ZnO_*x*_ samples under 1 V bias and UV stimulus.

The fLDW process allows for the production of sensors with tailored responses to external stimuli, by guiding electron movement on rGO sheets, as shown in Fig. 2a. A breakdown of the mechanisms involved is briefly discussed in ESI Section 1.†Fig. 2b highlights the sensing mechanism for n-type and p-type metal-oxide sensors (MOSs). In LiG/MO_*x*_ sensors, the response is governed by the conductive properties of the LiG film, thereby influencing the overall sensing behavior.

XRD spectra reveal that increased laser energy results in decreased crystallinity of the LiG film. In Fig. 2c, the LiG film displays prominent peaks characteristic of graphene oxide at approximately 14° and 22° (*d* = 0.822 and 0.432 nm) at low energy. Conversely, with the high laser energy density of fabrication, these GO peaks become broader. Both films exhibit a graphite peak at 26.6° (*d* = 0.335 nm), albeit less noticeable at higher laser energy. The broader and smoother peaks suggest a greater presence of amorphous structures. Initially exhibiting n-type conductivity, GO transforms p-type rGO through the reduction of oxygen-donating groups, thereby inducing p-type conductivity.^22^

To evaluate the efficacy of fLDW in printing n-type and p-type LiG films, a hot probe test^58^ was conducted on samples produced at varying laser energy densities. Below 2 J mm^−3^, the process primarily deposits an n-type film; while between 2 and 2.5 J mm^−3^, the LiG exhibits p-type characteristics. The conductivity nature can be controlled by modifying laser energy density as presented in Fig. 2d. This conductivity switch aligns with the surface functional groups on the LiG surface identified through XPS C 1s scans in Fig. 2e. At lower laser energy densities, the sample shows a higher ratio of electron-donating groups, marked by a higher ratio of C=O groups with a greater likelihood of lone pairs to donate.^59^ A noticeable transition occurs at around 2 J mm^−3^, where the ratio of C–N groups increases, exerting electron-withdrawing effects because the nitro groups, known for stable π bonds with electronegative atoms, contribute to this transition.^22,57,60–62^ XPS spectra analysis concludes that fLDW effectively controls LiG film conductivity through surface modification of functional carbonyl and oxygen groups.^63^

A LiG/ZnO_*x*_ sensor was fabricated, and its response to UVA (1.085 W m^−2^) under 1 V bias is shown in Fig. 2f. Upon activating the UV source at 2 seconds, the n-type LiG/ZnO_*x*_ sensor's normalized current gain increases by a factor of 1.7, while the p-type sensor's normalized current gain decreases by an equivalent factor. Thus, the response fLDW sensors can be effectively tuned positively or negatively by controlling the conductivity nature of the LiG films.

### 3.3 Effects of sp^2^ carbon hybridization in LiG films

**Fig. 3:**
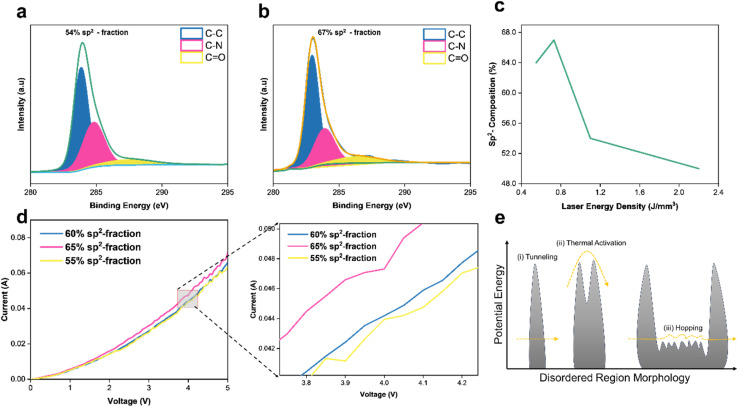
(a) XPS C 1s scan of a high energy density 1.2 (J mm^−3^) LiG film. (b) XPS C 1s scan of low energy density 0.8 (J mm^−3^) LiG film. (c) Laser energy density of fLDW process *vs.* sp^2^ fraction. (d) *I*–*V* curve of LiG films with increasing sp^2^ C–C fraction. (e) Electron hopping mechanism on GO surface morphology.

In addition to controlling conductivity nature, fLDW has the capability to modify the morphology of disordered regions in the LiG film, thereby influencing the mobility of charge carriers, as illustrated in Fig. 3. ESI Section 2† details electron transport mechanisms on rGO films. Fig. 3a and b demonstrates that laser energy density regulates sp^2^ carbon formations on LiG, which directly influences electron hopping. Three discernible peaks are highlighted: sp^2^-carbon (peak C–C), defect/sp^3^-carbon (C–N), and bonds of carbonyls (C=O). The sp^2^-carbon fraction, indicative of higher Hall mobility, is determined by multiplying the percentage of total carbon from C 1s bond values identified by XPS spectra. fLDW conducted at lower energy densities result in a higher fraction of sp^2^ carbons as evidenced by XPS C 1s spectra of LiG films induced at 1.2 J mm^−3^ and 0.8 J mm^−3^ respectively (and compiled on the *I*–*V* curve in Fig. 3c). Fig. 3d confirms a higher fraction of sp^2^ carbon corresponds to lower resistance due to a greater electron movement facilitated by an increase in the hopping Hall mobility of charge carriers.^64^ At a 4 V bias, the LiG film with a 60% sp^2^ fraction exhibits a 4.9% higher current output than the 55% sp^2^ fraction film. Thus, fLDW holds promise for tuning electrical properties in LiG/MO_*x*_ sensors by controlling the fraction of sp^2^ carbon. Usually, the reduction of rGO increases the sp^2^ carbon fraction, reducing resistivity and enhancing charge carrier mobility^61,65^ due to range hopping, as shown in Fig. 3e. The low potential barriers between crystalline sp^2^ domains and disordered regions offer lower resistance and greater electron mobility.^66^

### 3.4 Effects of LiG film crumpling

**Fig. 4:**
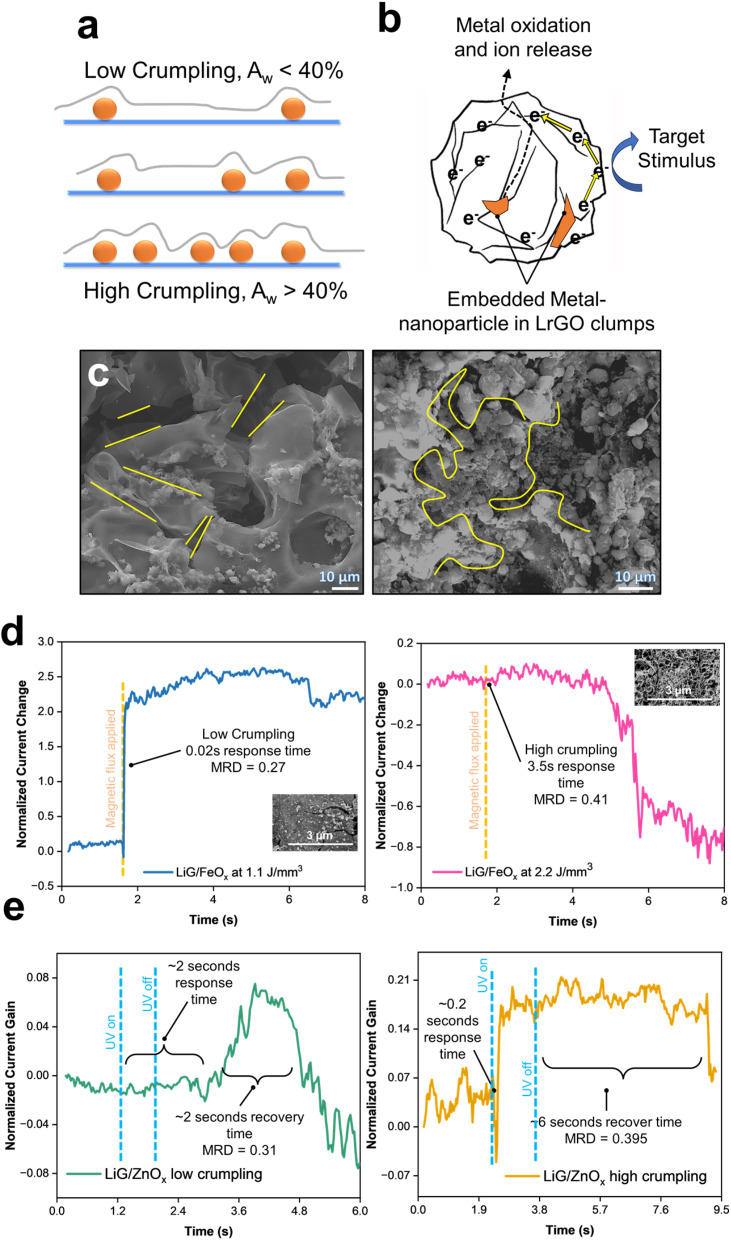
(a) Nano-particle induced crumpling of rGO mechanism in LiG/MO_*x*_ films. (b) Sensing mechanism in crumpled rGO sheets with MO nanoparticles. (c) SEM image of LiG crumpling after nanoparticle deposition, low laser energy density (left), and high laser energy density (right). Time *vs.* change in current output of (d) LiG/FeO_*x*_ and (e) LiG/ZnO_*x*_ with different rGO crumpling.

During step two of laser scanning (Fig. 1a), the initial LiG layer could undergo variable deforming forces caused by the plasma plume as it travels across the LiG surface inducing localized strains^67,68^ leading to crumpling of LiG films. Detailed mechanisms are outlined in ESI Section 3.† This crumpling process can influence the electronic properties of tunable LiG/MO_*x*_ sensors by inducing magnetic fields, altering local potentials, and impacting electron mobility across the 3D graphene matrix.^19,69^ As illustrated in Fig. 4a, the concentration and localization of nanoparticles can influence the crumpling affinity of LiG sheets. The presence of defects and electronegative nanoparticles leads to stronger folding due to the smaller distance between graphene and adhesion sites facilitated by van der Waals forces.^69–71^ The crumpling in the porous LiG film could affect the metal-oxide nanoparticle sensing process, as shown in Fig. 4b. When metal oxides transfer electrons to graphene, equalizing Fermi levels and facilitating their transfer to internal carbon structures, it will enhance the oxygen reduction on the outer surface. This reaction would be accelerated if the transport through metal oxides is inherently slow.^72,73^ Conversely, folded porous LiG sheets with high crumpling encase metal-oxide nanoparticles physically to separate oxide species from the metal-oxide cation release location, leading to a decelerated electron transport.^69,72,73^ Thus, the overall effect of crumpling is determined by metal-oxide composition.

Fig. 4c illustrates the difference between a low-crumpled (left) and highly-crumpled (right) LiG film. On the left, the stacking of LiG sheets is evident with nanoparticles on the surface. However, as the laser energy is increased, the LiG sheets start coalescing to form distinct clumps. The bonds between MO_*x*_ and LiG bridge the electron transfer channels and tighten the connection between them, creating an affinity for metal agglomeration, increased structural stability, and defect zones which attract rGO sheets towards centralized locations.^74–76^ Nonetheless, quantifying the extent of crumpling and correlating it to a specific laser energy density is somewhat difficult due to the complexities during fLDW deposition. To address the complexity, an image processing technique using shapelet functions called the response distance method^77^ was modified to process SEM images of the LiG films to determine the mean response distance (MRD) which provides a linear correlation to crumpling. The shapelet-based code^78^ and detailed explanation of crumpling mechanisms with XPS scan data correlations are provided in ESI Section 3.† Table S1† also summarizes the fractions of bonds between metal-oxide and LiG, and corresponding MRD (crumpling factors) for all the films at various laser energy densities.

Fig. 4d emphasizes the effect of crumpling on LiG/FeO_*x*_ sensors. Different LiG/FeO_*x*_ samples were fabricated at different laser energy densities, resulting in distinct levels of crumpling. The sample fabricated at 1.1 J mm^−3^ showed minimal crumpling (MRD = 0.27), in which an immediate response to the stimuli was achieved with a current gain factor of 2 under a magnetic flux of 10^−4^ T. On the other hand, the high porosity and crumpling sample (MRD = 0.41) at 2.2 J mm^−3^ exhibited a significant increase in response time of 3.5 s from exposure to an equivalent magnetic field strength.

Furthermore, recovery time in LiG/ZnO_*x*_ was observed under a short UV pulse of 1.085 W m^−2^ of 0.5 seconds, see Fig. 4e. The low crumpled LiG/ZnO_*x*_ film (MRD = 0.31) took 2 seconds to respond to the UV pulse and displayed a 2 seconds recovery time after. In contrast, the highly-crumpled LiG/ZnO_*x*_ film (MRD = 0.395) responded immediately within 0.2 seconds and required 6 seconds to recover. Interestingly, high crumpling has opposite effects on response time for FeO_*x*_ and ZnO_*x*_. Thus, the net effect of decelerating and accelerating nanoparticle oxidation, through rGO crumpling, is dependent on the metal-oxide species.^69,79^

### 3.5 Effects of metal oxide nanoparticles

**Fig. 5:**
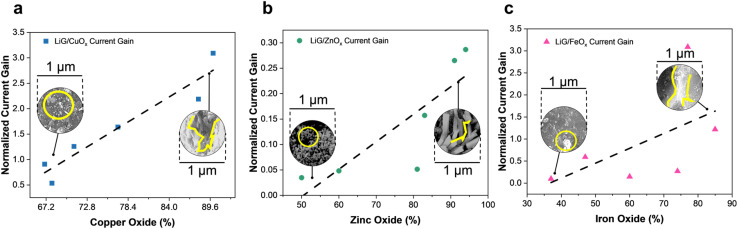
Normalized current gain *vs.* metal oxide percentage extrapolated from XPS spectra of, (a) copper oxide film (b) zinc oxide and (c) iron oxide.

The tunable band gap and the ability of 2D/3D graphene material to act as a molecular scaffold for metal/metal oxide nanoparticles can tune and enhance the electrical properties of resistive sensors.^7,55,57,72^ ESI Section 4† details the resistive sensing mechanism of the oxides. Fig. 5 presents the tunable responsivity of different metal oxides laser-treated through fLDW. The oxide content was extrapolated from the XPS spectrum of metal in the films. In Fig. 5a, the current gain of LiG/CuO_*x*_ sensors is shown to increase with CuO_*x*_ content. The normalized current gain is defined as the change in sensor output of an n-type-LiG/CuO_*x*_ film under exposure to visible light of 1.5 W m^−2^ for 5 seconds. Furthermore, as highlighted in Fig. 5a, SEM images showed agglomerated CuO_*x*_ on the LiG surface. The flexibility of the fLDW process allows replacing CuO_*x*_ with ZnO_*x*_ by only changing the precursor contents during step two deposition. Similar to CuO_*x*_ a higher concentration of ZnO_*x*_ results in a greater normalized current gain when exposed to UV light of 1.085 W m^−2^ for 5 seconds as illustrated in Fig. 5b because ZnO_*x*_ relies on the photoelectric effect.^80^ Lastly, the fLDW process was applied to deposit iron oxides on LiG as well. An n-type-LiG/FeO_*x*_ film under exposure to a magnetic flux of 10^−4^ T for 5 seconds exhibits greater response at higher FeO_*x*_ concentration due to particle agglomeration and greater contact points between graphene/metal-oxide composite, see Fig. 5c, similar to the other reported transition metal oxides.^81,82^

**Fig. 6:**
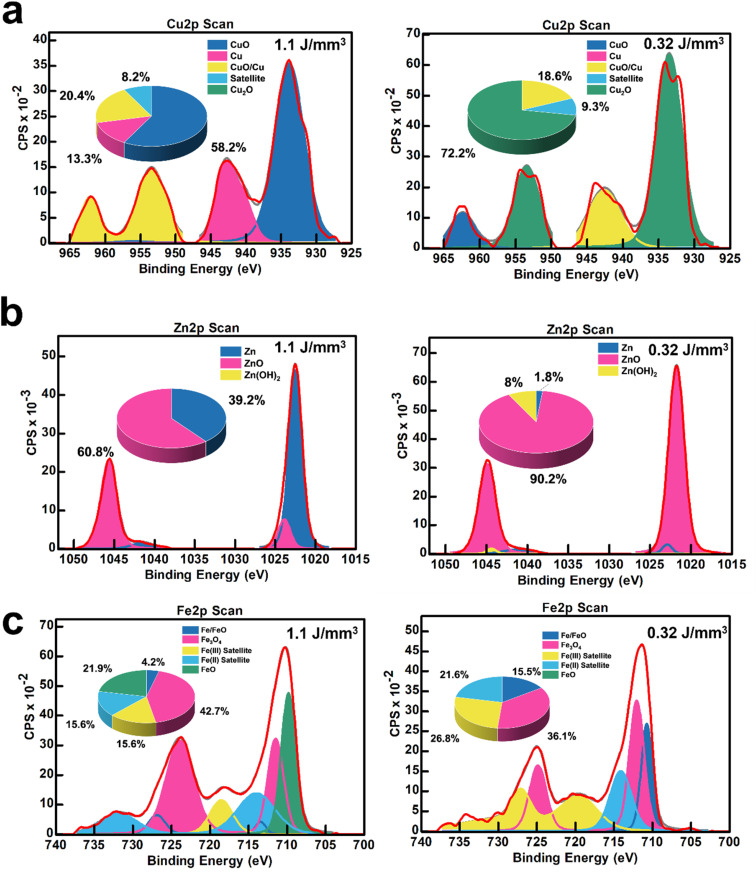
(a) XPS Cu 2p scans of LiG/CuO_*x*_ sensor films with high CuO and Cu_2_O content; (b) XPS Zn 2p scans of LiG/ZnO_*x*_ sensor films with different ZnO content; and (c) XPS Fe 2p scans of LiG/FeO_*x*_ sensor films with different FeO_*x*_ compositions.

Fig. 6 highlights the ability to tune nanoparticle composition (metal and metal oxide atomic percentages) by adjusting the laser energy density (0.32 and 1.1 J mm^−3^). XPS Cu 2p scans in Fig. 6a establish that fLDW can deposit a CuO-concentrated nanoparticle blend of 58.2 at% CuO, 20.4 at% CuO/Cu, and 13.3 at% Cu at high laser energy, which can be tuned to deposit a more Cu_2_O concentrated (72.2 at%) composition at low energy. The level of reduction of the Cu ions in the precursor solution to Cu^2+^ and Cu^+^ can be effectively controlled by varying the laser energy density.^25^ fLDW successfully deposited ZnO_*x*_ nanoparticles composed of 60.8 at% ZnO and 39.2 at% Zn metal at high laser energy density which could be changed to a composition of 90.2 at% ZnO and 8 at% Zn metal at low laser energy density, as shown in Fig. 6b. Therefore, the normalized current gain of a UV LiG/ZnO_*x*_ sensor can be effectively optimized by changing the surface concentration of ZnO in the LiG-ZnO_*x*_ matrix. LiG/FeO_*x*_ sensors, as presented in Fig. 6c, show low energy density fabrication has a film composition breakdown of 15.5 at% Fe/FeO and 36 at% Fe_3_O_4_, whereas, the nanoparticles have a greater Fe_3_O_4_ composition of 42.7 at% and FeO/Fe metal of 4.2 at% at high energy density.

**Fig. 7:**
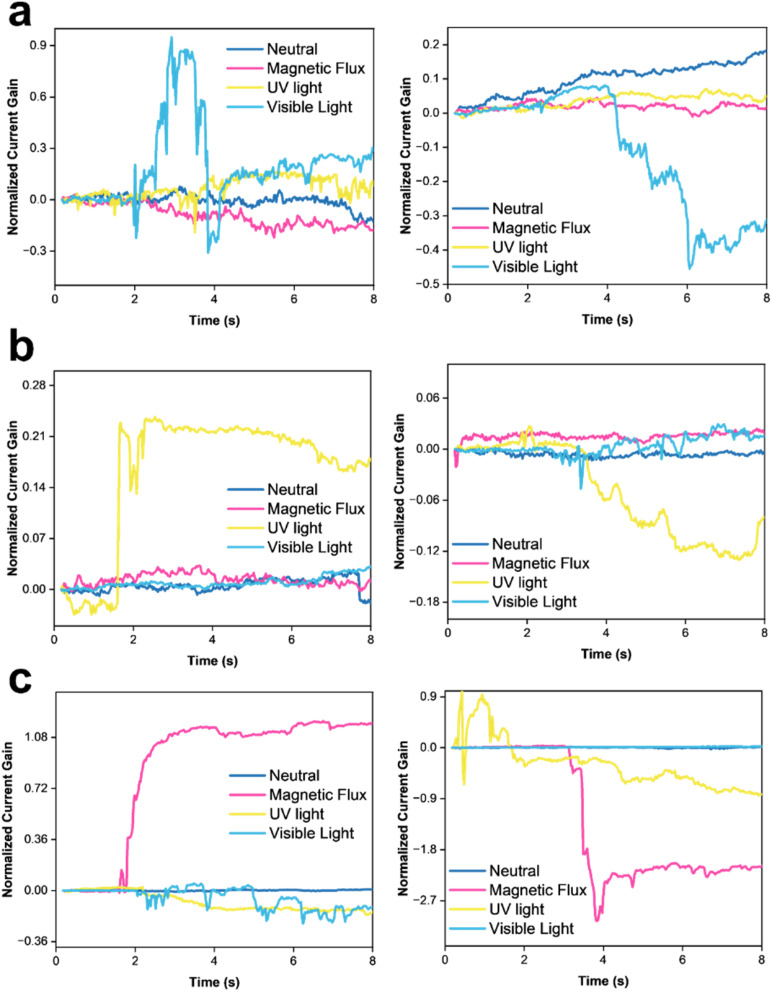
*I*–*V* curves of time *vs.* normalized change in current of (a) n-type LiG/CuO_*x*_ sensor (left) and p-type LiG/CuO_*x*_ (right), (b) n-type LiG/ZnO_*x*_ sensor (left) and p-type LiG/FeO_*x*_ sensor (right), and (c) n-type LiG/FeO_*x*_ sensor (left) and p-type LiG/FeO_*x*_ sensor (right).

To identify the selectivity of the metal oxide nanoparticles in LiG/MO_*x*_ sensors, different stimuli were used, including a magnetic flux of 10^−4^ T, UV light of 1.085 W m^−2^, and visible blue light of 1.5 W m^−2^. For n-type LiG/CuO_*x*_, the presence of CuO nanoparticles selectively reacts with blue visible light, demonstrating a normalized current gain maximum of +0.9 as presented in Fig. 7a. Subjection to the remaining stimuli was confined to a normalized gain of ±0.3. However, interestingly, the resistance immediately decreased to its neutral state despite continuous light exposure. The gradual change in sensor output over time could be due to aging, temperature, humidity, or other environmental factors.^83,84^ A p-type LiG/CuO_*x*_ sensor reacted similarly but with a decrease in current output with a normalized current gain of −0.4. Other stimulants also did not indicate a significant current gain, suggesting an adequate selectivity of light for LiG/CuO_*x*_ sensors.

In Fig. 7b, a n-type LiG/ZnO_*x*_ sensor showed a normalized current gain of 0.21 to UV light but an indifference to the other stimuli nearing a 0 normalized current gain. The p-type LiG/ZnO_*x*_ presents a comparable result with a normalized gain of −0.12 under UV light and <0.05 for the rest of the stimuli. The UV light selectivity of LiG/ZnO_*x*_ is greater than the visible light selectivity of LiG/CuO_*x*_.^85^

Fig. 7c shows an n-type LiG/FeO_*x*_ exhibited a great normalized current gain of 1.08 to a magnetic field. Contrasting the other transition metal oxides, the aligned FeO_*x*_ could retain the alignment after the removal of the external magnetic field.^7,86^ The p-type LiG/FeO_*x*_ also behaves similarly with a −2.7 normalized current gain.

### 3.6 Flexible sensor demonstration

**Fig. 8:**
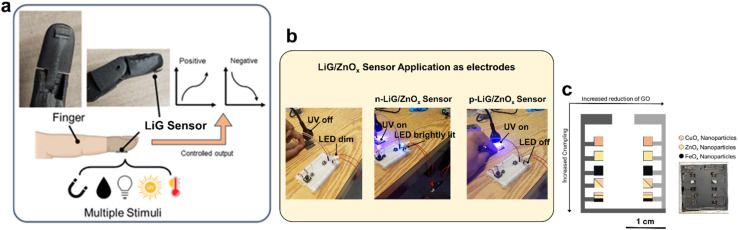
Demonstration of different sensors. (a) Schematic of LiG/MO_*x*_ sensor application on a mechanical finger. (b) Applying as electrode for LiG/ZnO_*x*_ sensors. (c) Schematic and photo of a multifunctional LiG/MO_*x*_ sensor grid.

The LiG/MO_*x*_ sensors are designed for fingertips, as illustrated in Fig. 8a. These sensors, using PI film as a substrate, can contour to meet the weight, flexibility, durability, multi-deformation, stretching, twisting, bending, and compression requirements of the biological body. Fig. 8b demonstrates the effective applicability of LiG/MO_*x*_ films as laser-treated-electrodes. In a series circuit, where the LiG/ZnO_*x*_ acts as an electrode, the tunable current gain under UV light is evident. For the n-type LiG/ZnO_*x*_ film, when subjected to a UV light stimulus of 1.085 W m^−2^, the sensor's resistance will decrease. As a result, the LED in the circuit will increase in brightness due to the higher conductivity. Conversely, when a p-type LiG/ZnO_*x*_ is exposed to the same UV illumination, the sensor's resistance will increase, lowering the current enough to turn off the LED light. Lastly, Fig. 8c illustrates a proof-of-concept sensor grid that combines all 3 metal oxides to highlight the versatility and tunability of fLDW. The LiG layer mimics electrodes and placements of the positive and negative terminals will modify electron transport through the sensor. Effectively, increased reduction of GO and crumpling can contour sensor outputs based on probe placements, while nanoparticle components can provide sensing of selective stimuli.

#### 3.6.1. LiG temperature sensors

**Fig. 9:**
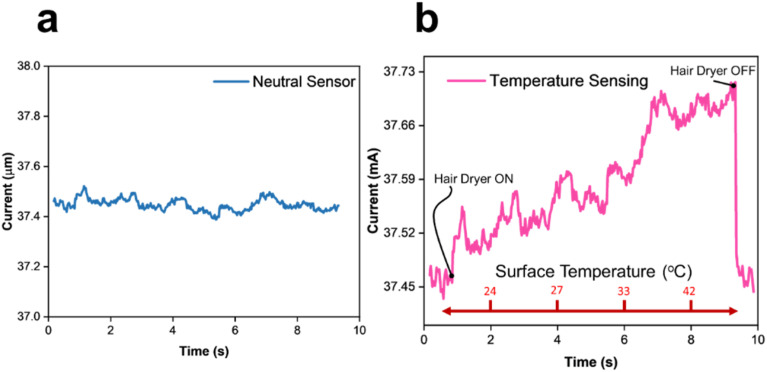
(a) LiG sensor 1 V bias without stimuli (b) temperature response of LiG sensor.

The next figures highlight the strength, and more importantly, the exceptional tunability of the LiG/MO_*x*_ sensor. First, Fig. 9a provides the current output of a LiG sensor, without metal/metal-oxide nanoparticles, under 1 V bias. There is a minor oscillation in the current output over time without any stimuli. The LiG shows a stable increase in conductivity with temperature growth, as illustrated in Fig. 9b. The innate temperature sensing of graphene membranes due to the density of states (DoS) near Fermi energy allows the LiG sensor to respond to temperature variation.^66^ The temperature gradient adds either electrons or holes to the graphene channel, leading to an increase in conductivity. It shows great stability and recovery within the temperature range of 27–45 °C. Furthermore, the sensor recovers to its original resistive state in 0.08 seconds after turning off the heat source. The LiG exhibits applicability for skin-inspired sensors to monitor body temperature.^47^

#### 3.6.2. LiG/ZnO_*x*_ UV sensors

**Fig. 10:**
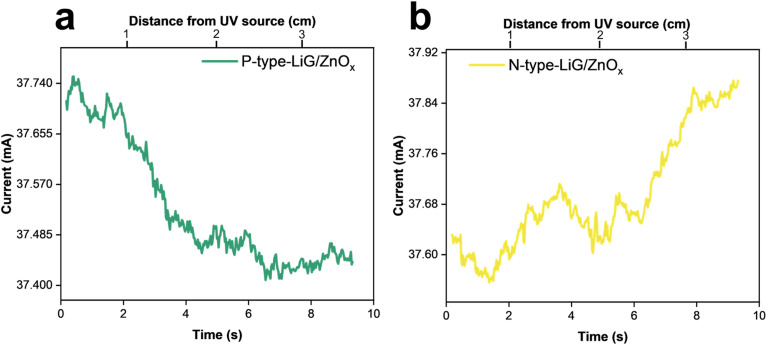
UV responses of (a) p-type and (b) n-type, LiG/ZnO_*x*_ sensors.

A p-type LiG/ZnO_*x*_ and n-type LiG/ZnO_*x*_ sensor were attached to a mechanical finger. The response of the sensor was gauged at variable distances from a UV light source of 1.085 W m^−2^ intensity. fLDW highlights stability in sensor fabrication, as both sensors exhibited a consistent resistance gradient, as illustrated in Fig. 10a and b. The p-type sensor's current output decreased by 0.34 mA, whereas the n-type has an output increased by 0.32 mA, at a maximum distance of 3 cm from the UV source. The test mimics by what means LiG/MO_*x*_ sensors, produced by fLDW, can be used to create tunable positive and negative sensors for fingertips to be used for proximity sensing.

#### 3.6.3. LiG/CuO_*x*_ light sensors

**Fig. 11:**
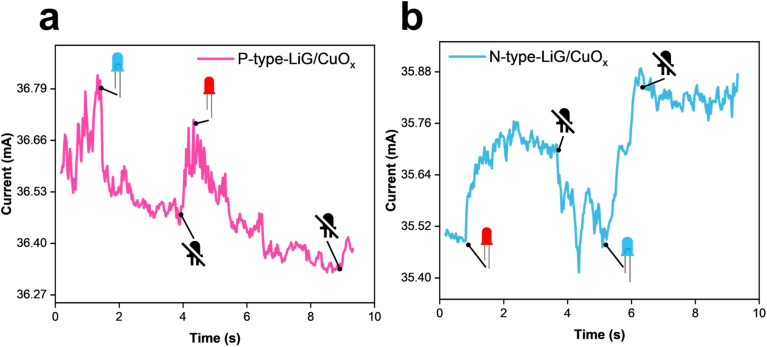
Visible light responses of (a) p-type and (b) n-type LiG/CuO_*x*_ sensors.

Fig. 11a and b presents the visible light response of p-type and n-type LiG/CuO_*x*_ sensors. The p-type sensor current output decreased by 0.26 mA when subjected to blue light and by 0.18 mA under red light. The sensor showed selectivity to LEDs of different intensities. Similarly, the n-type sensor showed an increase in current output of 0.32 mA under blue light and 0.26 mA under red light. The sensors showed a low recovery time of < 0.2 s after the first LED was turned off; however, after the second LED, the sensor retained its new resistive state – a factor of rGO electron saturation, the recovery time was greatly affected by multiple sensor inputs.^30,60,87^ This demonstrates the capability of fLDW to create light sensors that can distinguish between lights of different intensities.

#### 3.6.4. LiG humidity sensors

**Fig. 12:**
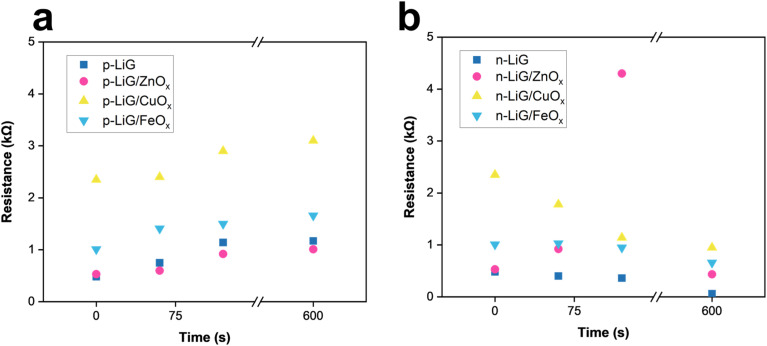
Humidity responses of (a) p-type and (b) n-type LiG/MO_*x*_ sensors.

Additionally, the LiG/MO_*x*_ sensors were placed in a 95% humidity chamber for extended durations and their changes in resistance. P-type LiG/CuO_*x*_ (in Fig. 12a) showed the greatest resistance increase from 2.2 kΩ to 3.1 kΩ after 10 minutes in the humidity chamber. Similar to the visible light sensing of LiG/CuO_*x*_, the conductivity nature of the LiG dictates whether the sensor increases or decreases in resistance when affected by humidity.^26^ Likewise, the n-type-LiG/CuO_*x*_ decreases in resistance the largest from 2.3 kΩ to 1.1 kΩ after 10 elapsed minutes as shown in Fig. 12b. The other nanoparticles had a minimal response to humidity.

#### 3.6.5. LiG sensor performance and durability tests

Table 1 compares the performance of LiG/MO_*x*_ sensors with similar flexible sensors, demonstrating that the LiG/MO_*x*_ sensors offer a comparable response. For example, the UV response of LiG/ZnO_*x*_ films shows a current of 7.13 μA at 1 V, which is comparable to the 5.5 μA at 1.5 V observed for ZnO/ethyl cellulose films. However, the key advantage of the fLDW technique is its greater customizability of the physical properties of the sensor. For instance, the responsivity of the LiG/ZnO_*x*_ UV sensor can be tuned between 0.2 and 6 s, while an Al-nitride-nanowire UV sensor operates within a narrower range of 0.27 to 0.41 s. This flexibility in adjusting sensor response times and response intensity offers significant advantages in designing sensors for specific applications.

**Table 1:** Performance evaluation of LiG sensors and other flexible sensors

Type of sensor/device	Materials	Evaluation metric	Performance	Ref.	This work
Photosensor	Perylene/graphene	Resistance in dark	∼5 MΩ	88	∼4 MΩ LiG/CuO_*x*_
UV photodetector	Al-nitride-nanowire	Responsivity	0.27–0.41 s to UV light illumination	89	0.2–6 s based on LiG/ZnO_*x*_ crumpling
Flexible photodetector	Ti_3_C_2_T_*x*_	Photoresponse	3.06 mA W^−1^	90	0.21 mA per W LiG/CuO_*x*_
UV sensor	ZnO/ethyl cellulose	Resistance	5.5 μA at 1.5 V	91	7.13 μA at 1 V LiG/ZnO_*x*_
Light photodetector	Graphene/amorphous Ga_2_O_3_	Photocurrent	∼10^5^ photo-to-dark ratio	92	10–10^4^ photo-to-dark LiG/CuO_*x*_

Table 2 shows the resilience of wearable LiG sensors; compared to various flexible photodetectors, LEDs, and gas sensors to evaluate their durability under challenging conditions *via* bend tests and performance assessment after 200 days in air. Bend tests were conducted with a bending radius of approximately 4.02 mm, resulting in a calculated surface strain of 5.001.^97^ Impressively, the LiG/MO_*x*_ sensors showed minimal degradation after 200 days in the air, maintaining normalized current gains at 70–80%, response times at 91–98%, and recovery times at 91% of initial values. This consistent performance was observed across all metal oxides integrated into the graphene matrix, suggesting the protective nature of dense porous films produced from fLDW against residual oxidation of metal species in reactive environments,^98^ thereby preserving normalized response over extended periods.

Endurance test summary of LiG sensors and other flexible sensors

**Table:** 

Type of sensor/device	Materials	Evaluation metric	Performance change after endurance tests (% initial)	Ref.
Temperature sensor	Ni/NiO	Temperature coefficient of resistance (TCR)	21.9% after 8000 bend tests	93
Wearable LED	Ag NPs/PDMS	Luminance	82% after 300 bend cycles	94
Photosensor	Perylene/graphene	Resistance	93% after 1000 bend cycles	88
UV exposure sensor	Ortho-nitro benzyl	Yellowness index change (Y_1_)	88% after 3.8 mm bend for 1 hour	95
Gas sensor	rGO/cotton-yarn	Normalized response (*R*_n_)	94% after 1000 bend cycles	96
			98% after 7 days of washing	
UV photodetector	Al-nitride-nanowire	Responsivity	75% after 100 bend cycles	89
Flexible photodetector	Ti_3_C_2_T_*x*_	Photoresponse	80% after 1000 bend cycles	90
UV sensor	ZnO/ethyl cellulose	Resistance	91% after 100 bend cycles	91
Light photodetector	Graphene/amorphous Ga_2_O_3_	Photocurrent	80% after 1000 bend cycles	92

**Table:** 

Type of sensor/device	Materials	Evaluation metric	Performance change after endurance tests (% initial)				Ref.
				200 days in air	200 days w/1000 bend cycles	200 days w/8000 bend cycles	
Variety of resistive sensors	LiG/MO_*x*_	Various	LiG/CuO_*x*_ normalized current gain	80%	50%	46%	This work
			LiG/CuO_*x*_ response time	98%	93%	94%	
			LiG/ZnO_*x*_ normalized current gain	70%	40%	43%	
			LiG/ZnO_*x*_ response time	91%	90%	81%	
			LiG/FeO_*x*_ recovery time	91%	90%	81%	

However, a discrepancy was noted in performance degradation between normalized current and response/recovery times following bend tests. After 1000 bend cycles, normalized current gain decreased to 40–50%, further declining to 43–46% after 8000 bend cycles. In contrast, recovery and response times only degraded to 90–93% after 1000 bend cycles and 81–94% after 8000 bend cycles. The initial steep degradation in performance after 1000 cycles suggests that the TI surface layers weakly bonded on devices are unable to withstand bend cycles, leading to fractures and defects with low conductivity.^99^ Conversely, the minimal reduction in performance after 1000 cycles suggests that the thicker LiG film, strongly bonded to the PI film, is more resilient to mechanical stressors. After the initial drop, the performance remains relatively stable. Furthermore, the programmable morphology, oxidation, crumpling, and doping characteristics of LiG produced by fLDW are robust, as these properties changed minimally in air and after bend cycles.^87,100^

## 4 Conclusion

In conclusion, a novel fLDW process was used to rapidly fabricate and combine reduced graphene and metal oxide nanoparticles to produce different sensors for the large-scale production of flexible electronics. Selectivity to humidity, visible light, UV light, and magnetic fluxes was demonstrated by LiG/CuO_*x*_, LiG/ZnO_*x*_, and LiG/FeO_*x*_, respectively. The deposition mechanism, enabled by laser parameter modifications, allowed for simultaneous surface engineering of the LiG, including sp^2^-carbon fractionization, 3D crumpling of rGO sheets, and for modifying the composition of metal oxides to tune the electrical properties of LiG/MO_*x*_ sensors. The normalized current gains were controllable by adjusting metal oxide nanoparticle compositions and sp^2^ carbon fraction, ranging from −2.7 to 2.7. Response times were effectively modified by inducing crumpling structures on rGO sheets, varying from 0.02 to 3.5 seconds. Similarly, recovery times could be manipulated within the range of 2 to 6 seconds. The versatility of fLDW is further underscored by the fabrication of a flexible device featuring tunable LiG/MO_*x*_ sensors on polyimide substrates. These sensors are designed to conform to the requirements of biological bodies, displaying potential for personalized and adaptable sensing applications. Additionally, the programmable properties exhibited remarkable endurance even after 200 days in air and through numerous extended bend cycles. This advancement not only addresses challenges associated with traditional production methods but also offers exceptional controllability in selectivity, response time, recovery time, reproducibility, and stability.

## Data availability

Data for this article is available at Zenodo at https://doi.org/10.5281/zenodo.14299824.

## Conflicts of interest

The authors declare that they have no conflicts of interest regarding the publication of this article.

## Supplementary Material

NA-007-D4NA00463A-s001

NA-007-D4NA00463A-s002

NA-007-D4NA00463A-s003

NA-007-D4NA00463A-s004

NA-007-D4NA00463A-s005

NA-007-D4NA00463A-s006

NA-007-D4NA00463A-s007
