# Supplementary figures and images for: Laser writing of metal-oxide doped graphene films for tunable sensor applications
Source: Nanoscale Adv. 2024 Dec 10;7(3):766–83. doi: 10.1039/d4na00463a (PMC11632522; doi:10.1039/d4na00463a)

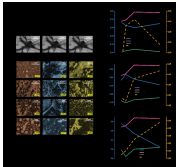

Supplement: NA-007-D4NA00463A-s006 [file NA-007-D4NA00463A-s006.pdf]
