# Supplementary material for: Laser writing of metal-oxide doped graphene films for tunable sensor applications
Source: Nanoscale Adv. 2024 Dec 10;7(3):766–83. doi: 10.1039/d4na00463a (PMC11632522; doi:10.1039/d4na00463a)
Supplement: NA-007-D4NA00463A-s007 [file NA-007-D4NA00463A-s007.pdf]

Supplementary Information (SI) for Nanoscale Advances.
This journal is © The Royal Society of Chemistry 2024

Laser Writing of Metal-Oxide Doped Graphene Oxide Films for Tunable Sensor Applications

*Shasvat Rathod, Monika Snowdon, Matthew Peres Tino, Peng Peng**

Corresponding author email: peng.peng@uwaterloo.ca

Centre for Advanced Materials Joining, Department of Mechanical and Mechatronics Engineering, University of Waterloo, 200 University Avenue West, Waterloo, Ontario, N2L 3G1, Canada

SECTION 1: fLDW TECHNICAL PROCESS EVALUATION

SECTION 1a: Graphene film characteristics

Supplementary Fig. S1 demonstrates the key stages of the laser writing process used to generate LiG from a polyimide (PI) film. Initially, laser irradiation induces the cleavage of C-N and C-O bonds in the imide groups (-CO-N-CO-) of the polyimide's chemical structure, leading to its transformation into graphene-like materials as shown in Fig. S1a. During the laser writing process, the controlled movement of the laser beam across the PI film enables precise patterning of localized regions, forming LiG[1], as exhibited in Fig. S1b.

The cross-sectional view of the PI film during irradiation, in Fig. S1c, reveals the emergence of different thermal zones due to the laser-material interaction. At the surface, a plasma region forms, where the high-energy laser beam ablates the material. Surrounding the plasma is splashed material, representing ejected fragments from the PI film. Beneath this layer, the hot-formed zone experiences C-N bond breaking, resulting in the formation of graphene structures. Below the hot-formed zone lies the hot molten zone, where the material becomes molten due to the laser's intense heat. Finally, there is the heat-affected zone, where the material is thermally influenced but remains structurally intact. The ejected splashed material coalesces to form porous graphene on the PI substrate[2]. The laser energy density directly impacts the size of the plasma region and the amount of splashed material, offering precise control over the characteristics and quality of the resulting graphene films. For fLDW setup, the range of viable laser energy density thresholds were identified to be 0.4 – 2.5 J/mm^3. Below 0.4 J/mm^3, the laser energy was too low to ablate the surface to produce LiG, and above 2.5 J/mm^3, the produced graphene was ejected from the substrate, leaving a damaged PI film.

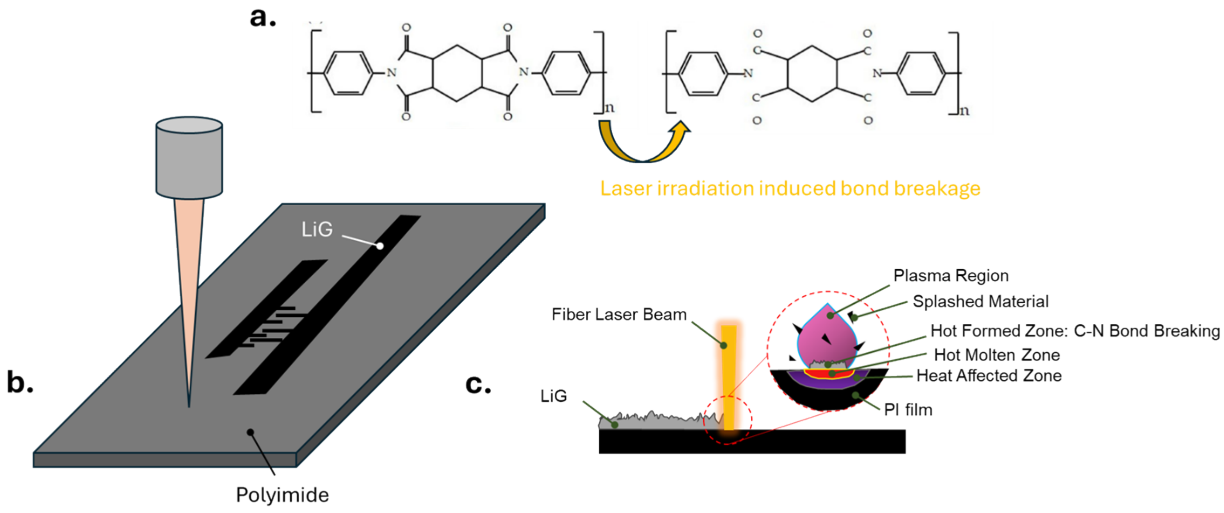

Fig. S1: fLDW of LiG production, laser writing overview (a) chemical reaction of PI film (b) laser writing patterning and (c) plasma plume effect on PI film.

Laser writing parameters such as scanning speed, laser power, and beam focus influence the laser energy density output of the fiber laser by affecting the plasma plume, as depicted in Fig. S1. Instead of examining the individual effects of each parameter on graphene film characteristics, the study considers the overall impact of laser energy density. Fig. S2 demonstrates that as laser energy density increases, both the sheet resistance and film width decrease, while the porosity of the film increases. The increase in porosity is attributed to higher energy causing more material to be ejected from the plasma plume region, leaving more holes in the laser-induced graphene (LiG) film. This material ejection reduces the film width as fewer sedimented materials remain. Interestingly, the decrease in sheet resistance is linked to the increased porosity, as the holes in the film allow ions to pass more easily through the graphene layers, enhancing conductivity[3]. The sheet resistance varies significantly, ranging from 625 to

125 Ω/sq., which correlates with porosity levels ranging from 57% to 80%, demonstrating a clear relationship between laser energy density and the electrical and structural properties of the graphene film.

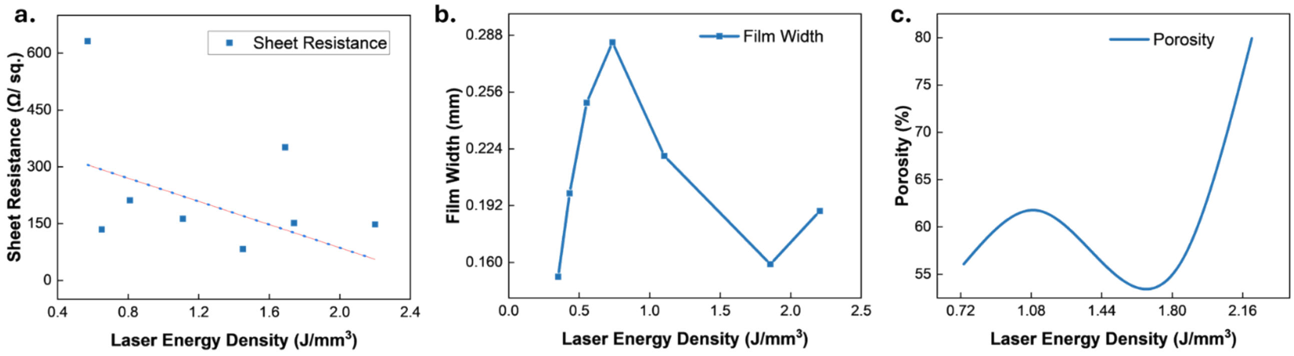

Fig. S2: Effect of writing parameters on (a) sheet resistance (b) film width and (c) porosity of graphene films.

SECTION 1b: Graphene-Metal Oxide film characteristics

Fig. S3 exhibits that graphene functionalization with metal oxides leads to a significant reduction in sheet resistance, attributed to the reduction of graphene oxide--double-bonded carbon in favor of forming metal–oxygen bonds. This functionalization increases the metal content within the graphene films, enhancing their conductivity. For example, introducing metals into the LiG reduces sheet resistance from 147 Ω/sq. in unmodified LiG to 47 Ω/sq. for LiG/FeO_x, 51 Ω/sq. for LiG/ZnO_x, and 82 Ω/sq. for LiG/CuO_x. This highlights the favorable impact of metal oxide incorporation in improving the electrical performance of graphene films by reducing resistance. Furthermore, higher laser energy densities increase metal-oxide bond formations indicating higher energy imparted in the process favors metal formation whereas lower energy density during fabrication favors oxides formations as shown by the reduction in C=O bonds[4].

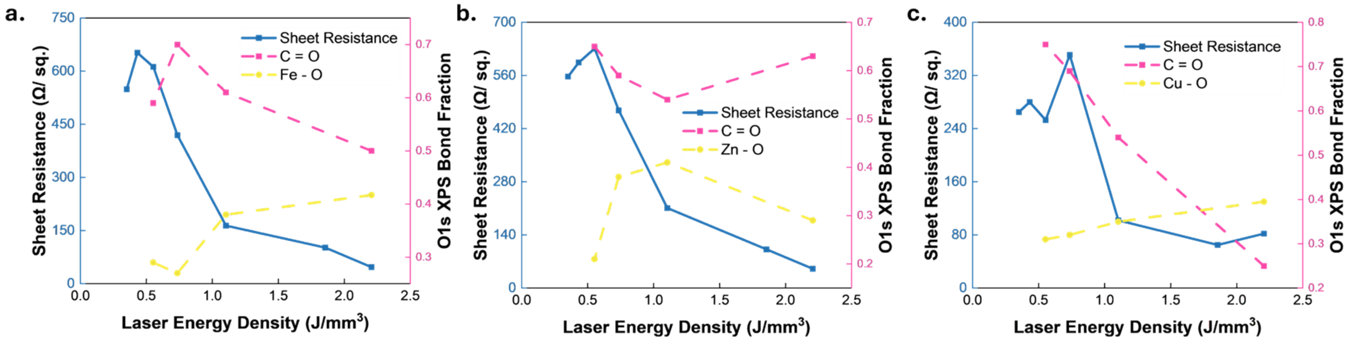

Fig. S3: Effect of Laser Energy Density on Sheet Resistance of Functionalized LiG with (a) FeO_x, (b) ZnO_x, and (c) CuO_x.

SECTION 2: ELECTRON TRANSPORT MECHANISMS

Optimization of charge transport in reduced graphene oxide is paramount for applications of rGO-based devices. Prior research has delved into the charge transport behavior of rGO, employing models such as "*phonon-assisted variable range hopping* (VRH)" and "*fluctuation-induced quantum tunneling added VRH*"[5–9] Furthermore, Baek et al. proposed that introducing potential barriers between crystalline sp^2 domains and disordered regions offers a more comprehensive explanation for rGO resistance, particularly at low temperatures[10]. Coupled with the ability of fLDW to produce LiG films with selective functional groups to change doping of carbon structures, there is promise to fabricate finely tuned graphene sensors with LDW[11].

SECTION 3: CRUMPLING CHARACTERIZATION WITH SHAPELETS

3a. SHAPELETS CODE

Shapelets were originally designed for the compression and reconstruction of images of galaxies[12]. Polar shapelets possess unique radial and rotational symmetry properties which have proven useful for analysis of circular/elliptical geometries. These shapelets were later reformulated by Suderman et al. to be used in order quantification for surface images of nanostructures with patterns present (stripe, square, hexagonal)[9,10]. Traditionally, shapelets are used as kernels and participate in convolution operations with target images (nanostructures). Computing the (discrete) convolution for a subset of shapelet functions at each pixel of an image yields a response vector. Typically, a reference region within the image that contains uniform pattern order (i.e. no defects/disorder) is determined by the user. This might be a region containing 10^2 or 10^3 pixels (and thus response vectors), which leads to high runtimes. Akdeniz et al. implemented k-means clustering, where k = 20, to condense the representation of this reference region into 20 response vectors[13].

The response distance for a response vector is computed as the minimum of all the Euclidean distances between itself and the 20 reference response vectors. A value of 0 would indicate the test response vector is clearly in the reference set. Larger values indicate more deviation from the reference set, suggesting local structural differences from the reference region (i.e. defects or disorder). Computing this process for each pixel (response vector) in an image is called the response distance method [ref: #9].

In this work, asimilar implementation of the response distance method from Suderman et al. is used but includes modifications to account for the lack of pattern symmetry present in the

microscopy images in this work (details are spared here). An open-source implementation of these shapelet-based methods[14] is available from www.github.com/uw-comphys/shapelets. . Using the response distance method in this manner allows for a general image processing technique to compare the topological structure between a reference and target image.

In this work, a higher-order subset of 30 Shapelets (m = [1, 30]) is used to obtain a response vector of length 30 for each pixel in an image. Note that the mth shapelet has m-fold symmetry. The choice to use this many shapelets is inspired by Tino et al., who realized that larger shapelet subsets, while not affecting computational runtime, reduced the noise associated with the response distance method .

A reference (non-doped) graphene sample image is used to compare topological differences to doped sample images (in contrast to a reference region within a single image). K-means clustering, with k = 50, was chosen to condense the numerous response vectors from the reference image into a condensed representation for computational efficiency. Then, response vectors are computed for a target image, and the response distance is computed for each response vector in the target image against the 50 reference response vectors. The mean response distance (MRD) is the sum of all response distances over the entire target image domain. It represents an overall suggestion of structural similarity between the reference image and the target image. As this value increases, it suggests that there are more local structural differences in the target image with respect to the reference. An example of the shapelets code output is presented in Fig. S4.

```
shas11@MSI:~/shapelets/examples/example_1$ shapelets config
Successfully loaded Cunode1.tif
Shape of image is: (698, 1233)
Image normalized to greyscale on [-1, 1]
Wavelength of image is 46.68 pixels
Convolution complete for shapelets m <= 18 before tolerance exceeded
kmeans successful with 20 centroids & distortion value of: 0.0931
Response distance 10% complete
Response distance 25% complete
Response distance 50% complete
Response distance 75% complete
Response distance 100% complete
Response distance runtime = 52.9 s
Figure Cunode1_response_distance_k20.png and Cunode1_response_distance_overlay_k20.png saved to /home/shas11/shapelets/examples/exa
mple_1/output/
```

Fig. S4: Python code output after using the shapelet-based algorithm

3b: DEFORMING FORCES ON LIG FILM LEADING TO CRUMPLING

The thin LiG film loses material as the second laser pass creates a micro plume. The ejected material, highly charged, is drawn to the newly sintered/nucleated nanoparticles, encasing them in crumples. Van der Waals interactions between graphene and metal nanoparticles, along with the low bending resistance of LiG films and high planar stiffness, drive this phenomenon[15–18]. This crumpling process can influence the electronic properties of tunable LiG/MO_x sensors by inducing magnetic fields, altering local potentials, and impacting electron mobility across the 3D graphene matrix[15,16].

Comparatively, FeO_x and CuO_x nanoparticles showed greater affinity for distortions, as it is more innately conductive than ZnO_x, and therefore is affected more physically and chemically by the folding LiG sheets. Furthermore, Fe metal has a smaller atomic radii and large concentrations of Fe metal in the rGO-matrix can provide smaller "jumps" in folds of the LiG sheets[19].

Fig. S5a illustrates the morphology changes of the LiG/MO_x films as laser energy density increases. The *mean response distances (MRD)* are provided on the bottom right of the SEM images processed by Shapelets.

LiG/CuO_x films exhibit an alternating pattern in MRD between 0.21 – 0.41, as laser energy density increases. Additionally, Shapelets functions show crumpling differs with metal-oxide selection as illustrated in Fig. S5b-d. Although there is no linear trend in MRD, XPS O1s scans of the film denote crumpling is increased with Cu-O-C covalent bonds formed between

CuO and rGO. Specifically, C=O peaks of oxygen functional groups on LiG (at 531.2, 531.9, and 533.8 eV) decreased with the addition of CuO_x nanoparticles from 0.87 to 0.54 after fLDW. As a result, Cu-O-C bonds increased accordingly, indicating that carbonyl and carboxyl of rGO react with Cu^{2+} ions, providing bridges for covalent bonds[20]. When the Cu-O-C bond fraction increases to 0.08, the crumpling of the LiG/CuO_x sheets is 0.41, whereas at low Cu-O-C bond fractions (0.04), the crumpling decreases to 0.28. Similar XPS O1s scan results are confirmed for LiG/FeO_x and LiG/ZnO_x films. Fig. S5b-d shows an average reduction in C=O bond fractions with the introduction of metal nanoparticles from 0.87 to 0.54 and 0.38, for LiG/FeO_x and LiG/ZnO_x, respectively. Furthermore, increased Fe-O-C and Zn-O-C covalent bond formations led to increased crumpling. The metal-oxide-carbon bonds between MO_x and rGO bridge the electron transfer channels and tighten the connection between the materials, creating an affinity for metal agglomeration, increased structural stability, and defect zones which attract rGO sheets towards centralized locations[19,21,22]. Table S1 summarizes the metal-oxide-carbon bond fractions and vector MRDs (crumpling factors) for all the films at various laser energy densities.

Table S1: Summary of Vector Differences (outputted by Shapelets) and corresponding XPS O1s scan bond fractions.

**Laser Energy Density (J/mm^3)**	**LiG**			**LiG/CuO_x**
	**C=O bond fraction**	**C=O bond fraction**	**M-O-C bond fraction**	**MRD (%)**
**2.206731**	0.87	0.25	0.02	0.29
**1.103365**	0.88	0.54	0.07	0.41
**0.735577**	0.71	0.69	0.04	0.38
**0.551683**	0.65	0.75	0.01	0.21

Laser Energy Density (J/mm^3)	LiG	LiG/FeO_x		
	C=O bond fraction	C=O bond fraction	M-O-C bond fraction	MRD (%)
**2.206731**	0.87	0.5	0.25	0.41
**1.103365**	0.88	0.61	0.21	0.38
**0.735577**	0.71	0.7	0.15	0.27
**0.551683**	0.65	0.59	0.19	0.29

Laser Energy Density (J/mm^3)	LiG	LiG/ZnO_x		
	C=O bond fraction	C=O bond fraction	M-O-C bond fraction	MRD (%)
**2.206731**	0.87	0.25	0.24	0.395
**1.103365**	0.88	0.54	0.2	0.35
**0.735577**	0.71	0.69	0.04	0.32
**0.551683**	0.65	0.75	0.18	0.31

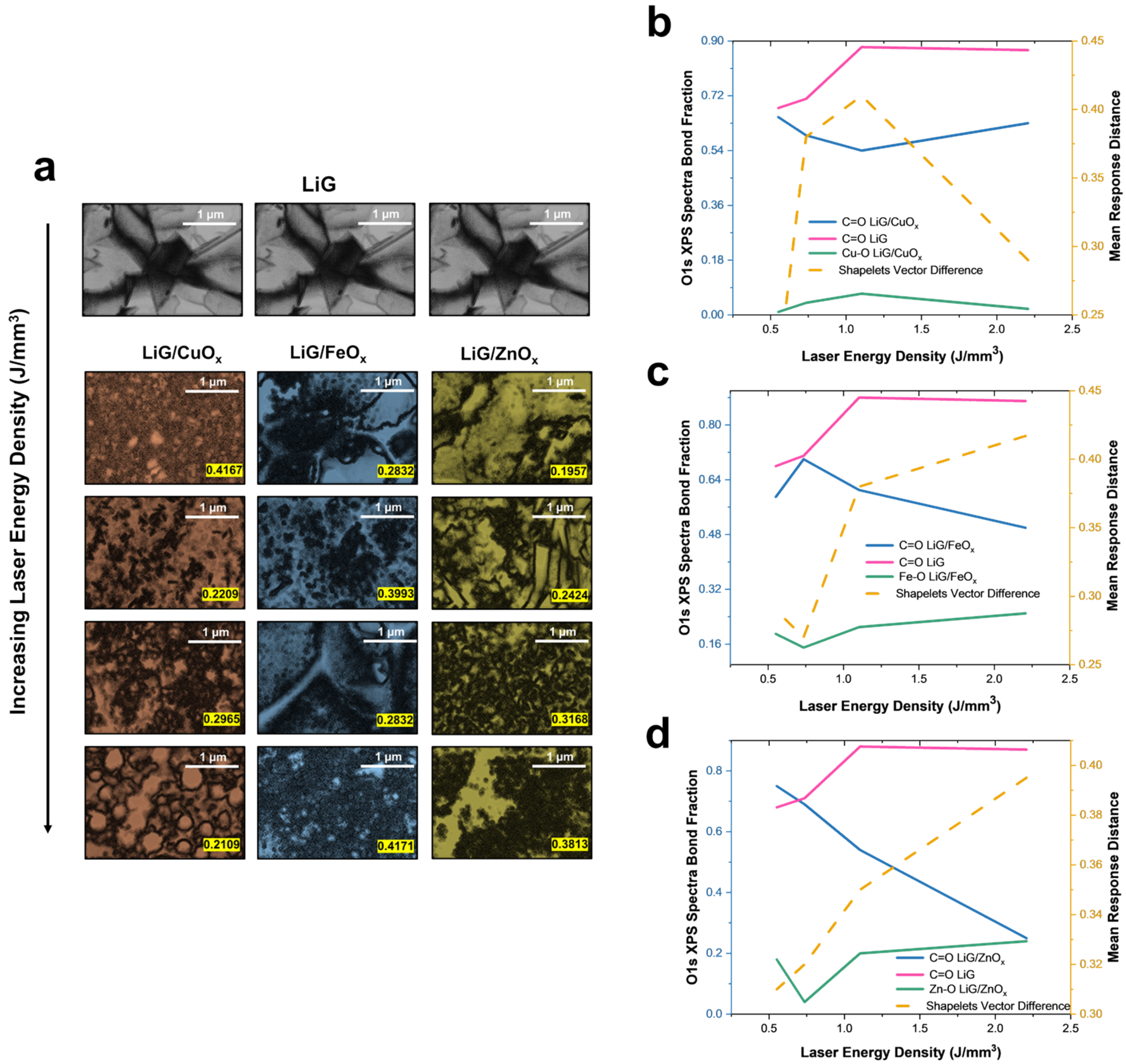

Fig. S5: Shapelets code output (a) Shapelet-based characterization of LiG/MOx films. (b) Laser energy density vs. Crumpling characteristics of LiG/CuOx films (c) Laser energy density vs. Crumpling characteristics of LiG/FeOx films (d) Laser energy density vs. Crumpling characteristics of LiG/ZnOx films

SECTION 4: RESISTIVE SENSING MECHANISMS

In n-type MOS, electrons are the majority charge transport, and the resistance of n-type MOSs decreases upon exposure to stimuli. On the other hand, in a p-type MOS sensor, holes are the majority charge transporters. In exposure to stimuli, the resistance of a p-type sensor increases as the concentration of holes increases due to the extracted electron. As extensively documented, copper oxide (CuO) is the ionic Cu state with the highest photodetection capabilities as a product of its narrow band gap of 1.2 eV, thus a larger concentration of CuO increases response sensitivity of visible light[23,24]. Agglomerated copper oxide particles, formed by the supplied laser heat at higher laser energy densities, encompass larger surface areas and grain sizes. The photo-carrier generation at the resistive core and adsorption response at the accumulation layer increase due to the increased volume fraction [20,25]. ZnO_x works as a UV light sensor through a similar mechanism. For FeO_x, although the sensing mechanism differs from CuO_x and ZnO_x, wherein, the unpaired electrons in the 3d subshell of ferric iron give rise to the magnetic attraction properties of the compound. Influenced by a magnetic field, the atoms are aligned complimentary to the field, reducing the resistance of the sensor, and thereby acting as FeO_x resistive sensors[26,27]. In totality, the resistive sensing magnitude of LiG/MO_x sensors are largely modifiable by increasing NP agglomeration on LiG sheets and altering NP composition.

REFERENCES

(1) Romero, F. J.; Gerardo, D.; Romero, R.; Ortiz-Gomez, I.; Salinas-Castillo, A.; Moraila-Martinez, C. L.; Rodriguez, N.; Morales, D. P. Supplementary Material: Comparison of Laser-Synthetized Nanographene-Based Electrodes for Flexible Supercapacitors. https://doi.org/10.3390/nano9091184.

(2) Bai, S.; Tang, Y.; Lin, L.; Ruan, L.; Song, R.; Chen, H.; Du, Y.; Lin, H.; Shan, Y.; Tang, Y. Investigation of Micro/Nano Formation Mechanism of Porous Graphene Induced by CO2 Laser Processing on Polyimide Film. *J. Manuf. Process.* **2022**, *84*, 555–564. https://doi.org/10.1016/J.JMAPRO.2022.10.037.

(3) *Porous structure gives graphene new superpowers*. https://www.nature.com/articles/d42473-024-00031-4 (accessed 2024-09-27).

(4) Muzyka, R.; Drewniak, S.; Pustelny, T.; Chrubasik, M.; Gryglewicz, G. Characterization of Graphite Oxide and Reduced Graphene Oxide Obtained from Different Graphite Precursors and Oxidized by Different Methods Using Raman Spectroscopy. *Materials (Basel).* **2018**, *11* (7), 1050. https://doi.org/10.3390/ma11071050.

(5) Liu, X.; Sui, Y.; Meng, C.; Han, Y. Tuning the Reactivity of Ru Nanoparticles by Defect Engineering of the Reduced Graphene Oxide Support †. **2014**. https://doi.org/10.1039/c4ra02900c.

(6) Kajen, R. S.; Chandrasekhar, N.; Pey, K. L.; Vijila, C.; Jaiswal, M.; Saravanan, S.; Ng, A. M. H.; Wong, C. P.; Loh, K. P. Charge Transport in Lightly Reduced Graphene Oxide: A Transport Energy Perspective. *J. Appl. Phys.* **2013**, *113* (6). https://doi.org/10.1063/1.4792042/371413.

(7) Bin Rahaman, A.; Sarkar, A.; Singha, T.; Chakraborty, K.; Dutta, S.; Pal, T.; Ghosh, S.; Datta, P. K.; Banerjee, D. Electrical Transport Properties and Ultrafast Optical Nonlinearity of RGO-Metal Chalcogenide Ensembles †. **2020**. https://doi.org/10.1039/c9na00728h.

(8) Yalcin, S. E.; Galande, C.; Kappera, R.; Yamaguchi, H.; Martinez, U.; Velizhanin, K. A.; Doorn, S. K.; Dattelbaum, A. M.; Chhowalla, M.; Ajayan, P. M.; Gupta, G.; Mohite, A. D. Direct Imaging of Charge Transport in Progressively Reduced Graphene Oxide Using Electrostatic Force Microscopy. **2015**. https://doi.org/10.1021/nn507150q.

(9) Pipinys, P.; Kiveris, A. Variable Range Hopping and/or Phonon-Assisted Tunneling Mechanism of Electronic Transport in Polymers and Carbon Nanotubes. *Cent. Eur. J. Phys.* **2012**, *10* (2), 271–281. https://doi.org/10.2478/S11534-012-0005-3/MACHINEREADABLECITATION/RIS.

(10) Saha, B.; Baek, S.; Lee, J. Highly Sensitive Bendable and Foldable Paper Sensors Based on Reduced Graphene Oxide. **2017**. https://doi.org/10.1021/acsami.6b10484.

(11) Rathod, S.; Snowdon, M.; Jones, J.; Zhang, K.; Peng, P. In Situ Rapid Fabrication of Graphene−Copper Heterojunctions Using Fiber Laser Direct Writing. *Cite This ACS Appl. Mater. Interfaces* **2023**, *15*, 57326. https://doi.org/10.1021/acsami.3c11552.

(12) Refregier, A. Shapelets - I. A Method for Image Analysis. *Mon. Not. R. Astron. Soc.* **2003**, *338* (1), 35–47. https://doi.org/10.1046/J.1365-8711.2003.05901.X/2/M_338-1-35-EQ055.JPEG.

(13) Akdeniz, T. J.; Lizotte, D. J.; Abukhdeir, N. M. A Generalized Shapelet-Based Method for Analysis of Nanostructured Surface Imaging. *Nanotechnology* **2018**, *30* (7), 075703. https://doi.org/10.1088/1361-6528/AAF353.

(14) Tino, M. P.; Abdulaziz, A. Y.; Suderman, R.; Akdeniz, T.; Abukhdeir, N. M. Shapelets: A Python Package Implementing Shapelet Functions and Their Applications. *J. Open Source Softw.* **2024**, *9* (95), 6058. https://doi.org/10.21105/JOSS.06058.

(15) Wang, Z.; Lv, X.; Chen, Y.; Liu, D.; Xu, X.; Palmore, G. T. R.; Hurt, R. H. Crumpled Graphene Nanoreactors. *Nanoscale* **2015**, *7* (22), 10267–10278. https://doi.org/10.1039/C5NR00963D.

(16) Hidayah, N. M. S.; Liu, W. W.; Lai, C. W.; Noriman, N. Z.; Khe, C. S.; Hashim, U.; Lee, H. C. Comparison on Graphite, Graphene Oxide and Reduced Graphene Oxide: Synthesis and Characterization. *AIP Conf. Proc.* **2017**, *1892* (1). https://doi.org/10.1063/1.5005764/965987.

(17) Yoon, Y.; Truong, P. L.; Lee, D.; Ko, S. H. Metal-Oxide Nanomaterials Synthesis and Applications in Flexible and Wearable Sensors. *ACS Nanosci. Au* **2022**, *2* (2), 64–92. https://doi.org/10.1021/ACSNANOSCIENCEAU.1C00029/ASSET/IMAGES/LARGE/NG1C00029_0014.JPEG.

(18) Gies, V.; Lopinski, G.; Augustine, J.; Cheung, T.; Kodra, O.; Zou, S. The Impact of Processing on the Cytotoxicity of Graphene Oxide. *Nanoscale Adv.* **2019**, *1* (2), 817–826. https://doi.org/10.1039/C8NA00178B.

(19) Gao, X.; Tang, Z.; Meng, M.; Yu, Q.; Zhu, Y.; Shen, S.; Yang, J. Synthesis of Crumpled SnO2/RGO Nanocomposites with 2D-in-3D Structure and High Performance. *Mater. Chem. Phys.* **2020**, *253*, 123298. https://doi.org/10.1016/J.MATCHEMPHYS.2020.123298.

(20) Gao, X.; Tang, Z.; Meng, M.; Yu, Q.; Zhu, Y.; Shen, S.; Yang, J. Synthesis of Crumpled SnO 2 /RGO Nanocomposites with 2D-in-3D Structure and High Performance. *Mater. Chem. Phys.* **2020**, *253*, 123298. https://doi.org/10.1016/j.matchemphys.2020.123298.

(21) Liu, Y. Z.; Chen, C. M.; Li, Y. F.; Li, X. M.; Kong, Q. Q.; Wang, M. Z. Crumpled Reduced Graphene Oxide by Flame-Induced Reduction of Graphite Oxide for Supercapacitive Energy Storage. *J. Mater. Chem. A* **2014**, *2* (16), 5730–5737. https://doi.org/10.1039/C3TA15082H.

(22) Gao, X.; Tang, Z.; Meng, M.; Yu, Q.; Li, J.; Shen, S.; Yang, J. Graphene Oxide Induced Assembly and Crumpling of Co3O4 Nanoplates. *Nanotechnology* **2020**, *31* (30), 305601. https://doi.org/10.1088/1361-6528/AB841F.

(23) Li, M. Y.; Yu, M.; Su, D.; Zhang, J.; Jiang, S.; Wu, J.; Wang, Q.; Liu, S. Ultrahigh Responsivity UV Photodetector Based on Cu Nanostructure/ZnO QD Hybrid Architectures. *Small* **2019**, *15* (28), 1901606. https://doi.org/10.1002/SMLL.201901606.

(24) Torres-Ochoa, J. A.; Cabrera-German, D.; Cortazar-Martinez, O.; Bravo-Sanchez, M.; Gomez-Sosa, G.; Herrera-Gomez, A. Peak-Fitting of Cu 2p Photoemission Spectra in Cu0, Cu1+, and Cu2+ Oxides: A Method for Discriminating Cu0 from Cu1+. *Appl. Surf. Sci.* **2023**, *622*, 156960. https://doi.org/10.1016/J.APSUSC.2023.156960.

(25) Deng, S.; Berry, V. Wrinkled, Rippled and Crumpled Graphene: An Overview of Formation Mechanism, Electronic Properties, and Applications. *Mater. Today* **2016**, *19* (4). https://doi.org/10.1016/j.mattod.2015.10.002.

(26) Koh, I.; Josephson, L. Magnetic Nanoparticle Sensors. *Sensors 2009, Vol. 9, Pages 8130-8145* **2009**, *9* (10), 8130–8145. https://doi.org/10.3390/S91008130.

(27) Urbanova, V.; Magro, M.; Gedanken, A.; Baratella, D.; Vianello, F.; Zboril, R. Nanocrystalline Iron Oxides, Composites, and Related Materials as a Platform for Electrochemical, Magnetic, and Chemical Biosensors. *Chem. Mater.* **2014**, *26* (23), 6653–6673. https://doi.org/10.1021/CM500364X/ASSET/IMAGES/LARGE/CM-2014-00364X_0036.JPEG.
