# Supplementary material for: Laser writing of metal-oxide doped graphene films for tunable sensor applications
Source: Nanoscale Adv. 2024 Dec 10;7(3):766–83. doi: 10.1039/d4na00463a (PMC11632522; doi:10.1039/d4na00463a)
Supplement: NA-007-D4NA00463A-s002 [file NA-007-D4NA00463A-s002.pdf]

Supplementary Information (SI) for Nanoscale Advances.
This journal is © The Royal Society of Chemistry 2024
